# Novel GLP-1 Fusion Chimera as Potent Long Acting GLP-1 Receptor Agonist

**DOI:** 10.1371/journal.pone.0012734

**Published:** 2010-09-15

**Authors:** Qinghua Wang, Kui Chen, Rui Liu, Fang Zhao, Sandeep Gupta, Nina Zhang, Gerald J. Prud'homme

**Affiliations:** 1 Division of Endocrinology and Metabolism, the Keenan Research Centre in the Li Ka Shing Knowledge Institute, St. Michael's Hospital, Toronto, Ontario, Canada; 2 Department of Physiology, University of Toronto, Toronto, Ontario, Canada; 3 Department of Medicine, University of Toronto, Toronto, Ontario, Canada; 4 Department of Laboratory Medicine, St Michael's Hospital, Toronto, Ontario, Canada; University of Bremen, Germany

## Abstract

GLP-1 has a variety of anti-diabetic effects. However, native GLP-1 is not suitable for therapy of diabetes due to its short half-life (t1/2<2 min). To circumvent this, we developed a long-lasting GLP-1 receptor agonist by the fusion of GLP-1 with human IgG2 Fc (GLP-1/*h*IgG2). ELISA-based receptor binding assay demonstrated that GLP-1/*h*IgG2 had high binding affinity to the GLP-1R in INS-1 cells (Kd = 13.90±1.52 nM). Upon binding, GLP-1/*h*IgG2 was rapidly internalized by INS-1 cells in a dynamin-dependent manner. Insulin RIA showed that GLP-1/IgG2 dose-dependently stimulated insulin secretion from INS-1 cells. Pharmacokinetic studies in CD1 mice showed that with intraperitoneal injection (i.p.), the GLP-1/*h*IgG2 peaked at 30 minutes in circulation and maintained a plateau for >168 h. Intraperitoneal glucose tolerance test (IPGTT) in mice showed that GLP-1/*h*IgG2 significantly decreased glucose excursion. Furthermore, IPGTT performed on mice one week after a single drug-injection also displayed significantly reduced glucose excursion, indicating that GLP-1/*h*IgG2 fusion protein has long-lasting effects on the modulation of glucose homeostasis. GLP-1/*h*IgG2 was found to be effective in reducing the incidence of diabetes in multiple-low-dose streptozotocin-induced type 1 diabetes in mice. Together, the long-lasting bioactive GLP-1/*h*IgG2 retains native GLP-1 activities and thus may serve as a potent GLP-1 receptor agonist.

## Introduction

Glucagon like peptide 1 (GLP-1) is a 30-amino acid peptide that is secreted from intestinal L-cells in response to nutrient ingestion and promotes nutrient absorption via regulation of islet hormone secretion [1]; [2]. Through activation of the GLP-1 receptor (GLP-1R), a G-protein-coupled receptor (GPCR), GLP-1 stimulates insulin secretion and suppresses glucagon secretion thereby lowering blood glucose in rodents as well as in humans [3]; [4]. In addition, GLP-1 increases insulin gene expression and upregulates insulin biosynthesis, via upregulation of the transcription factor pancreatic duodenal homeobox-1 (PDX-1). Within the pancreas, GLP-1 expands β-cell mass via promotion of β-cell growth and reduction of β-cell death in rodent models [5]; [6] and possibly in human as well [7]. Furthermore, GLP-1 also slows the rate of absorption of nutrients into the blood stream by reducing food intake and inhibiting gastric emptying [8]. Whole body GLP-1 receptor-null mice exhibit moderate glucose intolerance and disrupted islet architecture suggesting that GLP-1 receptor signaling in islets is required for normal function and development [9].

While the biological relevance and pathological impact is yet to be fully clarified, clinical studies have demonstrated that GLP-1 secretion is decreased in human subjects with diabetes, which highlights the potential use of GLP-1 as a therapeutic approach for type 2 diabetes. However, despite its numerous anti-diabetic functions, GLP-1 is rapidly degraded *in vivo*, with a half-life of <2 min, due to degrading enzyme DPP IV and rapid kidney clearance [10]. GLP-1 receptor agonists analogous to native GLP-1 have thus been developed as an alternative approach to increase GLP-1R activity in the treatment of type 2 diabetes. Exendin-4 (Ex-4), a lizard salivary gland peptide, that has high sequence homology to mammalian GLP-1 and is resistant to enzymatic degradation [8] has been approved for the treatment of type-2 diabetes since 2005 [11]. Other formulations using GLP-1 mimetics have been also developed to overcome the pharmacokinetic limitations of GLP-1 for the treatment of type 2 diabetes [11].

We have recently developed a platform for the production of GLP-1 fusion peptides consisting of GLP-1 or GLP-1 related molecules and immunoglobulin constant region or Fc domain (GLP-1-IgG-Fc) [12]; [13]. Various versions of GLP-1-IgG fusion proteins are designed based on different IgG sub-types or different IgG species for pre-clinic or clinic research. Using gene therapy strategy, we have demonstrated that a single intramuscular injection resulted in a persistent expression of GLP-1/IgG-Fc fusion protein in mice, which, as a results, improved insulin production and normalized glucose tolerance in T2D db/db mice [13] and reduced diabetes incidence in streptozotocin (STZ)-induced beta-cell injury T1D mouse model [12]. In the present study, we show that GLP-1/*h*IgG2, by fusion of GLP-1 with human IgG2 Fc, retains native GLP-1 activities. We demonstrate that GLP-1/IgG2 interacts with GLP-1R and stimulates insulin-secretion in insulin-secreting INS-1 cells, in a glucose-concentration dependent fashion. Upon binding, GLP-1/*h*IgG2 is rapidly endocytosed by INS-1 cells, in a dynamin-dependent manner. Pharmacokinetic studies in CD1 mice show that after a single intraperitoneal injection (i.p.), the fusion protein rapidly appears in circulation and remained at a high level for more than a week. The fusion protein has anti-diabetic effects, which is exemplified by its capacity in reducing the incidence of diabetes in multiple-low-dose streptozotocin-induced type 1 diabetes mouse model. Our results suggest that GLP-1/*h*IgG2 may serve as an alternative potent GLP-1 receptor agonist for the treatment of diabetes.

## Materials and Methods

### Animals

7-week-old CD1 mice (Charles River Laboratories, St Constant, QC, Canada) were housed under controlled temperature conditions and a 12-h light/12-h dark cycle in the St Michael's Hospital Animal facility with free access to normal rodent chow and water except where noted. All procedures were conducted according to protocols and guidelines approved by the Canadian Council of Animal Care and the St Michael's Hospital Animal Care committee. Before intervention, body weight, feeding blood glucose, intraperitoneal glucose tolerance testing (IPGTT), water consumption, food intake and urine volume were measured and a fasting blood sample was taken from the saphenous vein for serum insulin and glucagon measurement. For diabetes induction, 50 mg/kg body weight of STZ (Sigma Chemical Co., St Louis, MO, USA) was dissolved in 0.01 M cold citrate buffer (pH 4.5) immediately before intra-peritoneal injection (for 4 consecutive days). The development of diabetes was monitored by measuring blood glucose from the tail vein using Ascensia ELITE XL glucometer and Ascensia Elite blood glucose test strips.

### Cell culture

Rat insulinoma INS-1 cells (passage 50–65) were maintained in RPMI 1640 medium (Invitrogen, Burlington, ON, Canada) containing fetal bovine serum (10% v/v), 100 Units/ml penicillin G sodium, 100 µg/ml streptomycin sulphate, 55 mg/500 ml sodium pyruvate, 1.14 g/500 ml HEPES, and 1.7 µl/500 ml β-mercaptoethanol at 37°C in an atmosphere of humidified air (95%) and CO_2_ (5%). In studies involving serum-starvation, serum was replaced by 0.1% BSA in RPMI 1640 without glucose.

### Expression and purification of GLP-1/*h*IgG2

**Figure 1:**
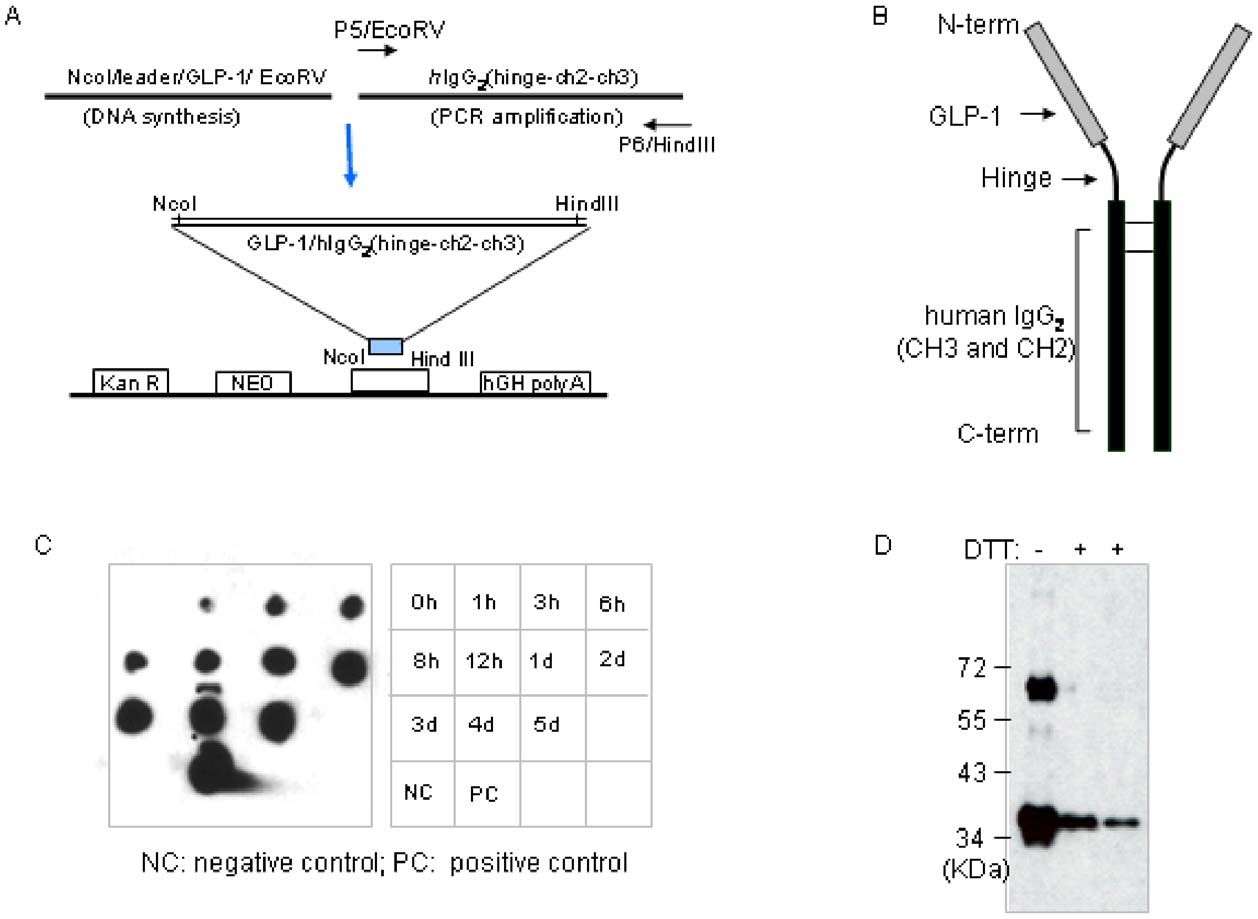
Construction of GLP-1/*h*IgG2 fusion protein. (A) Illustration shows that the cDNA encoding the fusion protein hGLP-1 chemically synthesized was ligated to a PCR-amplified cDNA fragment coding human IgG2 FC (hinge-ch2-ch3) and inserted into the NcoI and Hind III sites of a modified mammalian expression vector. (B) The secretable GLP-1/*h*IgG2-Fc fusion proteins are homodimeric. (C) Protein production efficiency was evaluated by the Dot blot with anti-human IgG antibodies using conditional medium from the CHO cells stably expressing the fusion expression. (D) Western blot shows that the GLP-1 fusion protein form stable dimer of molecular weight ∼70 kDa and monomer formation under reducing conditions.

A schematic (Figure 1A) shows that the cDNA encoding the fusion protein hGHRH/hGLP-1 was chemically synthesized, ligated to a PCR-amplified cDNA fragment encoding human IgG2 FC (hinge-ch2-ch3) and inserted into the NcoI and Hind III sites of the pAV0243 vector to generate GLP-1/*h*IgG-Fc/pAV0243. GLP-1/*h*IgG2 encoding DNA fragment was then amplified using pfu DNA polymerase (Fermentas, Glen Burnie, MD, USA). The GLP-1/*h*IgG2 gene was then double digested by restriction enzyme BamH I (New England Biolabs, Ipswich, MA, USA) and sub-cloned into a mammalian expression vector pMPGCR5 (a gift from Dr. R Gilbert, NRC Biotechnology Research Institute, Canada). For stable expression, CHO cells (a gift from Dr. R Gilbert) were expanded in CD-CHO complete medium (Invitrogen Life Science, Faraday Avenue Carlsbad, CA, USA) containing with 1×HT, 4 mM L-glutamine, then transfected with GLP-1/*h*IgG2-pMPGCR5 constructs. Stable expressing clones were selected by 600 mg/ml Hygromycin B (BioShop Canada Inc., Burlington, ON, Canada) screen and finally amplified into 500 ml CD-CHO complete medium in suspension culture at 225 rpm at 37°C until the cells density reached at 7×10^6^ cells/ml. The cultured CD-CHO medium was harvested, filtered and the GLP-1/*h*IgG2 fusion protein was purified by using Protein A Ceramic HyperD® F sorbent (Pall Corporation, Port Washington, NY, USA) and Immunopure Gentle Ag/Ab binding and elution buffers (Thermo Fisher Scientific, Rockford, IL, USA). Selected fractions were pooled, dialyzed into 1×PBS, pH 7.4 and stored at −80°C.

### Immunoblot

Purified GLP-1/*h*IgG2 fusion protein was resolved by10% SDS-PAGE and transferred to a PVDF membrane. The membrane was probed with goat-anti-human IgG antibodies (1∶3000, Southern Biotech, Birmingham, AL, USA) or anti-GLP-1 antibodies overnight at 4°C and detected using HRP-conjugated secondary antibodies (1∶5000, Jackson Lab, Bar Harbor, ME, USA) and visualized by enhanced chemiluminescence (GE Healthcare Bio-Sciences Corp., Piscataway, NJ, USA).

### Insulin secretion assay

INS-1 cells were plated in 24-well plates with a density of 2.5×10^5^ cells/well in RPMI 1640 medium containing 10% FBS. The following day, the medium was replaced with fresh KRB buffer devoid of glucose for 120 min. The cells were then treated with 2.8 or 16.8 mM glucose and various concentrations of purified GLP-1/*h*IgG2 fusion proteins in KRB buffer for 2 h. The insulin levels in conditioned KRB buffer were measured using a rat insulin RIA kit (Linco, St Charles, MO, USA) according to the manufacturers' instructions.

### GLP-1 receptor binding assay

The binding affinity of GLP-1/*h*IgG2 was determined in a setting of cellular ELISA. Briefly, INS-1 cells grown in 96 well plates (BD Biosciences) at roughly 95% confluence were rinsed with PBS and fixed with 4% paraformaldehyde (Thermo Scientific) for 10 min at room temperature and quenched for 5 min with 2% glycine in PBS, pH 7.5. For the binding capacity experiment, cells were incubated with logarithmic dilutions of GLP-1/Fc alone (1×10^−5^ to 1×10^−12^ M) for total binding or in combination with 10 µM GLP-1 (Abcam Inc, USA) for non-specific binding. For the competitive binding experiment, we used fixed GLP-1/Fc concentration (10 µM) and competed its binding with varying concentration of GLP-1, Exendin-4 and glucagon (Bachem Americas, Inc. USA) (1×10^−5^ to 1×10^−12^ M). After a 4-h incubation at 4°C in a final volume of 100 µl, excess GLP-1 receptor agonists were washed and the cells blocked with 5% BSA (BD Biosciences). Bound residual GLP-1/Fc was detected by goat anti-human IgG-Fc antibody (1∶4000, Southern Biotech) and detected by HRP-conjugated donkey anti-goat IgG (1∶5000, Jackson ImmunoResearch). Ortho-Phenylenediamine (OPD) substrate (Fisher Scientific) was added for enzymatic reaction and the colorimetric change was analyzed by reading absorbance at 490 nm in a Beckman microplate reader.

### Internalization Studies

Internalization of receptor-bound ligand was determined in INS-1 cells or INS-1 cells transfected with wild type or dominant negative dynamin constructs (kind gift of Dr. YT Wang, University of British Columbia, BC, Canada) using Lipofectamine 2000 according to manufacturer's instructions. In Brief, INS-1 cells were plated on poly-L-Lys coated 18 mm cover slip in 35 mm dishes at a confluence of 70%–80% in RPMI 1640 medium containing 10% FBS. The cells were switched into pre-cooled RPMI 1640 (10% FBS) medium containing 1 µM GLP-1/*h*IgG2 and incubated at 4°C for 30 minutes, then at 37°C for 0, 15, 60, and 90 minutes. The cells were then fixed with 3% paraformaldehyde for 10 min and blocked with 2% BSA in PBS containing 0.1% Triton X-100 at room temperature for 1 hour. The cells were then incubated with goat-anti-human IgG antibody (1∶500, Southern Biotech, Birmingham, AL, USA) and Cy3-conjugated anti-goat antibody (1∶500, Jackson Lab, Bar Harbour, ME, USA) consecutively. These cells were then stained with Top3 dye (1∶20,000) at room temperature for 10 minutes. The images were captured in a LEICA confocal microscope (DMIRE2).

### Pharmacokinetics of GLP-1/*h*IgG2 in mice

Male CD1 mice (n = 3) were intra-peritoneally injected with GLP-1/*h*IgG2 with a dose of 1 µg/mice. Serum samples were collected at 0, 0.5, 24, 96, 120, 168 and 192 hours after GLP-1/*h*IgG2 administration in DPP-IV inhibitors and aprotinin (10 µM and 50 KU/ml, respectively; Sigma Chemical Co., St Louis, MO, USA). The concentration of total GLP-1 was measured by using a total GLP-1 RIA kit (LINCO, St Charles, MO, USA) according to the manufacturer's instructions.

### GLP-1/*h*IgG2 stability assay

Serum samples were collected from mice, pooled and mixed, and kept on ice. Recombinant GLP-1 (Abcam, Inc) or GLP-1/*h*IgG2 fusion proteins were incubated with 50 µl serum samples at final concentration of 75 pmol. The mixtures were then incubated at 37°C for indicated time period allowing DPPIV-enzymatic reaction. The reactions were terminated by adding DPP-IV inhibitors and aprotinin (50 µM and 250 KU/ml, respectively) and un-degraded peptides were then determined by active GLP-1 ELISA kit (Linco, St Charles, MO, USA).

### Intraperitoneal glucose tolerance test (IPGTT), insulin tolerance test (ITT)

Male CD1 mice (n = 10) were fasted overnight for 15 h and were dosed intraperitoneally with GLP-1/*h*IgG2 (1 µg/mice) or saline as the control 30 minutes prior to the IPGTT. For IPGT, mice were given 1.5 g glucose/kg body weight (anhydrous dextrose; EMD Chemicals Inc., Gibbstown, NJ, USA) via intra-peritoneal injection. Blood was drawn from the tail vein and glucose levels were measured using a glucometer at 0, 10, 20, 30, 60 minutes after glucose administration. For ITT, mice were i.p. injected with insulin (2.0 U g/kg), blood glucose levels were measured at the indicated times.

### Statistical analysis

The data were analyzed and the binding curves were fitted by a one-site receptor model using Graphpad Prism 5.0 program. Briefly, Bmax and Kd of GLP-1/Fc were calculated by following the Specific-Nonspecifc binding algorithm for one site binding, using the formula: Specific binding  = Bmax * [L]/(Kd + [L]), where Bmax is the maximal binding at a given [L], [L] =  Ligand concentration. The IC50 for glucagon, GLP-1 and Exendin-4 were determined by the competitive binding for one site algorithm. All data were presented as mean ± SEM. Statistical analysis was performed using Student's t test. A p-value of less than 0.05 was considered to be statistically significant.

## Results

### GLP-1/*h*IgG2 fusion protein production

The expression constructs were engineered in an optimal balance of efficacy and safety. An illustration (Figure 1A) shows that the cDNA encoding the fusion protein hGLP-chemically synthesized was ligated to a PCR-amplified cDNA fragment coding human IgG2 FC (hinge-ch2-ch3) and inserted into the NcoI and Hind III sites of a mammalian expression vector to generate GLP-1/*h*IgG. The secretable GLP-1/*h*IgG-Fc fusion protein consisting of the active GLP-1 molecule [7]–[37] directly linked to the IgG-Fc encompassing the human IgG2 constant heavy-chain is shown in (Figure 1B). The linker between the two molecules is achieved by a design of the construct containing nucleic acids sequence code for the natural hinge region of the human IgG2, which provides flexibility facilitating the ligand-receptor binding [14]. Since the fusion junction does not contain an artificial linker and thus has minimized immunogenicity. The CHO cells stably transfect with the fusion expression vectors, in a suspension culture with serum-free chemical defined medium, have efficient production efficiency (Figure 1C). The fusion proteins are secreted as homodimers upon expression as determined by Western blot using anti-human IgG (Figure 1D) or anti-GLP-1 antibodies (not shown).

### In vitro characterization of GLP-1/*h*IgG2

**Figure 2:**
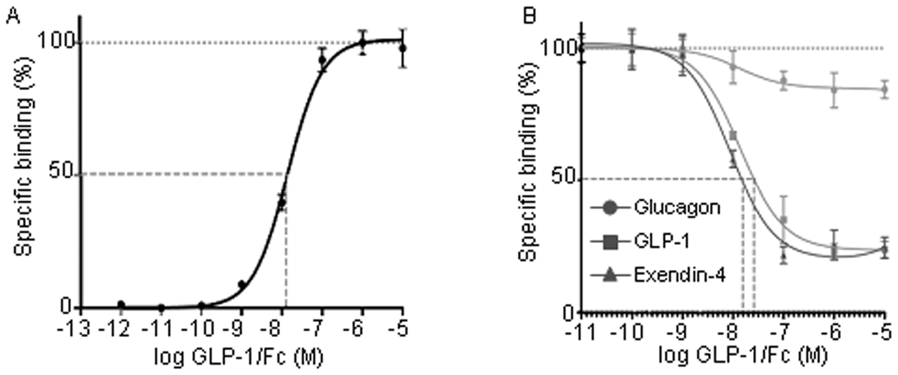
Assessment of the binding affinity of GLP-1/*h*IgG2. (A) Cellular ELISA based ligand-receptor binding assay shows the maximum binding of GLP-1/hIgG2 to INS-1 cells is ∼1 µM with Kd of 13.90±1.52 nM. (B). Competitive binding assay was performed in a reaction mixture containing 10 µM GLP-and its competitors Ex-4, GLP-1 and glucagon of varying concentration (1×10^−5^ to 1×10^−12^ M).

**Figure 3:**
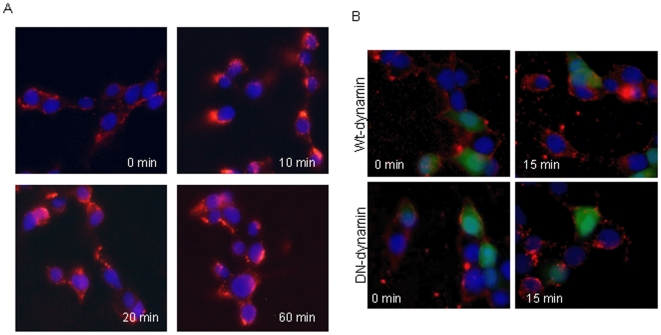
Endocytosis of GLP-1/*h*IgG2 is via a dynamin dependent manner. (A) Internalization of GLP-1/hIgG2-Fc in INS-1 cells (A) Cells were incubated with 1 µM GLP-1/*h*IgG2 at 4°C for 30 minutes, then switched to 37°C for 0, 10, 20 and 60 minutes. After fixation and blocking for non-specific binding, cells were then incubated with goat-anti-human IgG antibody (1∶500) followed by secondary Cy3-conjugated anti-goat antibody (1∶500) and Top3 dye (1∶20,000) for nuclei staining. The images were then visualized by a confocal microscope. (B) The internalization experiments were performed in cells co-transfected with either wild type dynamin or dominant negative dynamin and green fluorescent protein (GFP). 24 hours after transfection, internalization of GLP-1/*h*IgG2-Fc was conducted as described in (A). DAPI (1∶10,000) was used for nuclei staining and images were taken by a Nikon fluorescent microscope.

Using cellular ELISA based receptor binding assay we determined binding capacity of GLP-1/*h*IgG2 in INS-1 cells. The results showed that the binding of GLP-1/*h*IgG2 to INS-1 cells was fusion protein-concentration dependent, at a maximum binding of 1 µM GLP-1/Fc and with a K_d_ of 13.90±1.52 nM (Figure 2A). Results of competitive binding assays, where GLP-1/IgG2 concentration was fixed at 10 µM with varying concentration of Ex-4, GLP-1 and glucagon, showed 50% inhibition of the binding of GLP-1/*h*IgG2 to INS-1 cells (IC_50_) at 13.75±0.07 nM for native GLP-1 and 8.15±0.085 nM for exendin-4, respectively (Figure 2B). Glucagon could not compete with the fusion protein at the concentration range used. Internalization studies showed that GLP-1 receptors were rapidly internalized 10 min after stimulation, as demonstrated by an increase in the cytoplasmic GLP-1/*h*IgG2 staining (Figure 3A). However, the internalization was reduced in the INS-1 cells over-expressing dominant-negative dynamin by transfection, but not in the cells transfected with wild type dynamin (Figure 3B), suggesting that the internalization process of GLP-1/*h*IgG2-GLP-1R is partially dynamin-dependent [15].

**Figure 4:**
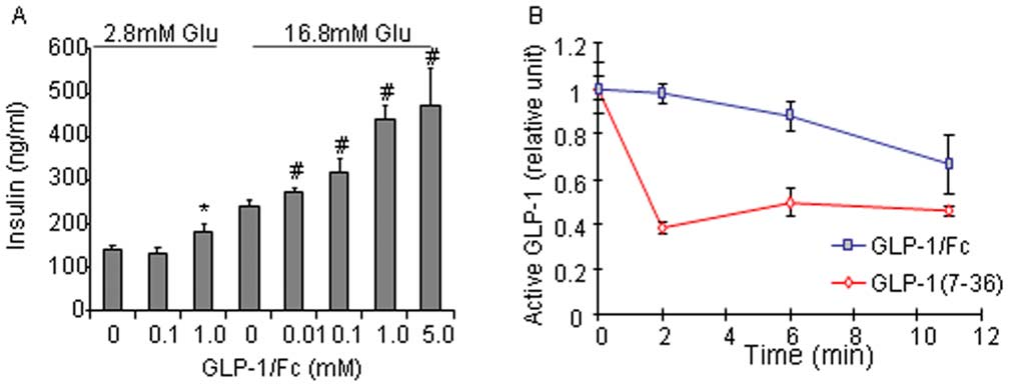
Stimulation of insulin secretion by GLP-1/*h*IgG2 in INS- cells. (A) Cells grown in 24-well plates were incubated with fresh KRB buffer devoid of glucose for 2×60 min. The cells were then treated with 2.8 or 16.8 mM glucose and various concentrations of purified GLP-1/*h*IgG2 fusion proteins in KRB buffer for 2 h. The insulin levels in the conditioned KRB buffer were measured using a rat insulin RIA kit. (B) Active GLP-1 was measured by active GLP-1 ELISA kit in the mouse serum samples incubated with recombinant GLP-1 (75 pM) or GLP-1/*h*IgG2 fusion proteins (75 pM) at 37°C for indicated time period. The reactions were terminated by adding excessive DPP-IV inhibitors and aprotinin.

Insulin secretion RIA showed that GLP-1/*h*IgG2 stimulated insulin secretion from INS-1 cells in a dose and glucose concentration dependent fashion (Figure 4A). To investigate whether GLP-1/*h*IgG2 fusion protein are more resistant to serum DPPIV, we conducted the stability assay using active GLP-1 ELISA kit. As shown (Figure 4B), both native GLP-1 and GLP-1/*h*IgG2 were degraded by serum DPPIV, however, the GLP-1 fusion protein displayed much slower decay rate, compared to the native GLP-1. These *in vitro* studies suggested that GLP-1/*h*IgG2 has biological relevance in insulin-secreting cells via activation of GLP-1 receptor, and that GLP-1 in the IgG fusion format is relatively resistant to the degrading enzyme(s).

**Figure 5:**
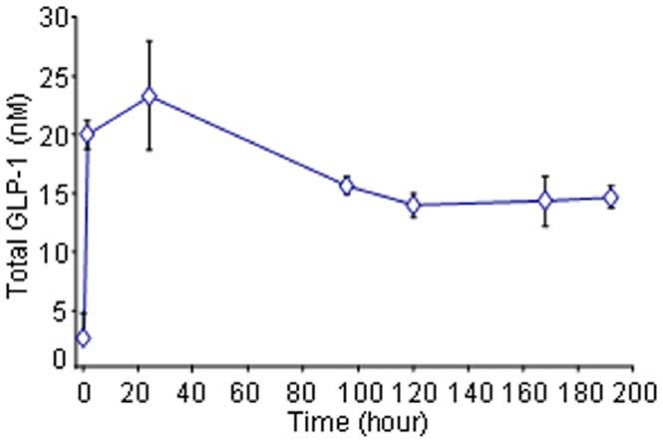
Pharmacokinetic study of GLP-1/*h*IgG2 fusion protein in CD1 mice. CD1 mice were i.p. injected by a single-dose of GLP-1/*h*IgG2 (1 µg/mouse). Blood samples were taken from tail vein at different time points and serum GLP-1 levels were measured by GLP-1 RIA kit.

### In vivo studies in mice

Pharmacokinetic data showed that 30–60 min after a single-dose administration in CD1 mice, circulating GLP-1/*h*IgG2 concentration was significantly increased as determined by GLP-1 RIA. The circulating fusion protein was found to plateau at 24-h and thereafter it gradually decreased but could still be detected 192-h after the single dose-injections (Figure 5).

**Figure 6:**
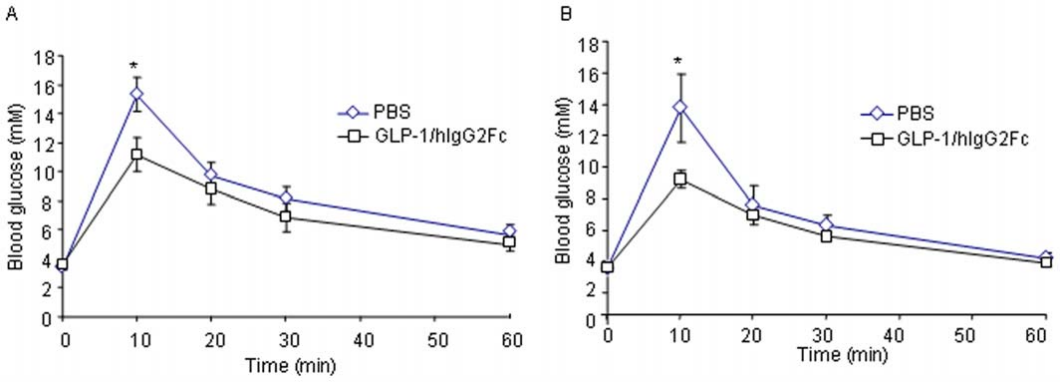
Intraperitoneal Glucose Tolerance Test (IPGTT) shows that GLP-1/*h*IgG2 improves glucose tolerance in CD-1 mice. After 16 h fasting, CD1 mice were i.p. injected with GLP-1/*h*IgG2 (1 µg/mouse). 30 min after the injection, IPGTT were conducted by i.p. injection of 1.5 g/kg of glucose and blood glucose levels were measured by a glucometer at 0, 10, 20, 30, 60 minutes after glucose administration. (B) 192-h after a single-dose injection of GLP-1/*h*IgG2 in CD1 mice, the mice were fasted for 16 h and IPGTT were conducted as described in (A).

To determine if the GLP-1/*h*IgG2 fusion protein has glucoregulatory effects *in vivo*, IPGTT was performed 30 min after the drug injection. As shown, GLP-1/*h*IgG2 reduced glucose excursion (Figure 6A). In order to examine if GLP-1/*h*IgG2 fusion protein has long-lasting effects on improving glucose tolerance, IPGTT was performed 192-h after a single-dose administration of GLP-1/*h*IgG2 in CD1 mice. As shown, while the fasting blood glucose levels were not different between GLP-1/*h*IgG2-injected mice and control mice, the drug-injected mice showed reduced glucose excursion (Figure 6B), suggesting that the fusion protein exerted long-lasting glucoregulatory effects in these mice.

**Figure 7:**
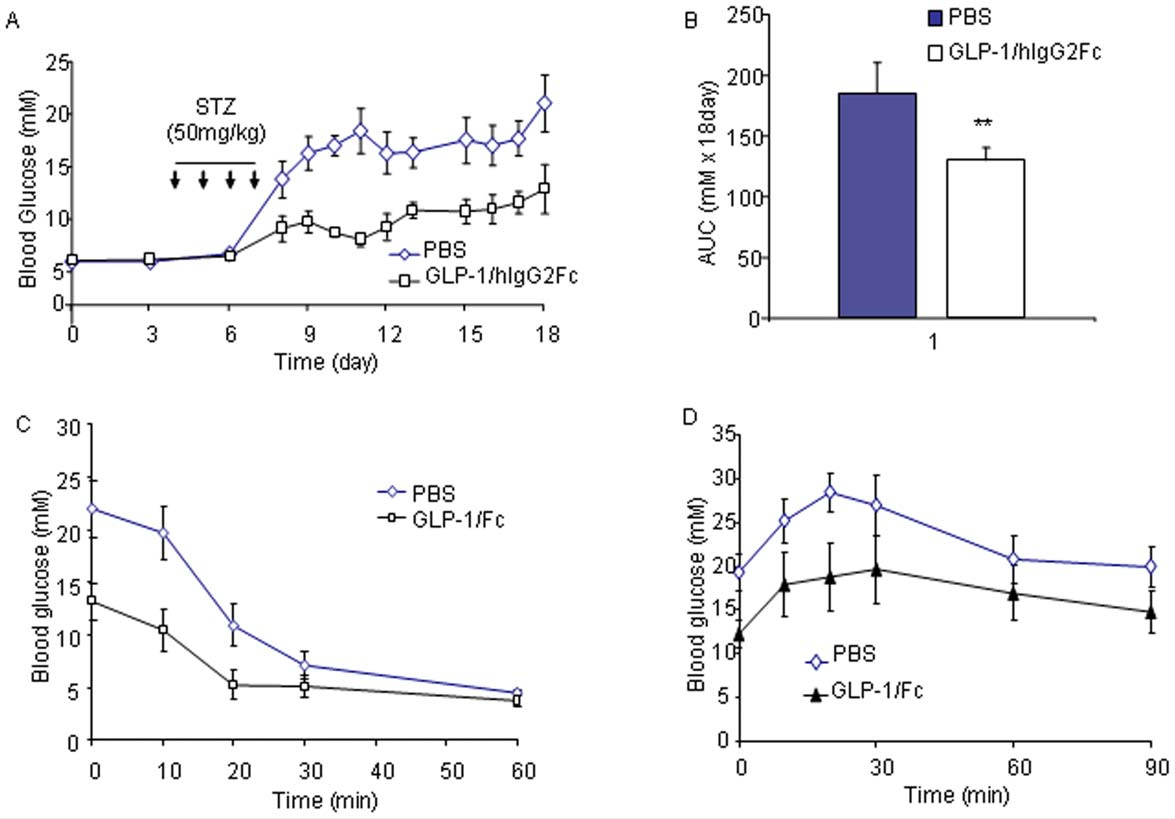
GLP-1/*h*IgG2 improves glucose regulation in MDSD mice. (A) The intraperitoneal injections of GLP-1/*h*IgG2 (1 µg/mouse) were made every three days during the course of experiment and the first drug injection was made 3 days prior to the multiple low dose streptozotozin treatment (50 mg/kg/day for 4 consecutive i.p. injections). Glucose levels were measured by a glucometer at indicated times. (B) The glucose levels were expressed as the area under the curve (AUC). (C) Insulin tolerance test (ITT) was conducted by injecting insulin (2.0 U/kg, i.p.) and blood glucose levels were measured at indicated times. (D) IPGTT was performed in MDSD mice treated with PBS or GLP-1/*h*IgG2-Fc at the end of the experiment as shown in (A).

We next assessed whether GLP-1/*h*IgG2 has anti-diabetic effects using multiple-low-dose streptozotocin-induced type 1 diabetes (MDSD) mice. The intraperitoneal injections of GLP-1/*h*IgG2 (1 µg/mouse) were made every third day during the feeding course and the first drug injection was made 3 days prior to the streptozotozin treatment. As shown, four consecutive intraperitoneal injections of low dose of STZ (50 mg/kg) induced overt diabetic hyperglycemia in all mice 5–7 days after the injections (Figure 7A; n = 5). In a contrast, the GLP-1/*h*IgG2-treated MDSD mice maintained relatively constant glucose levels, although higher than those measured at their earlier ages (Figure 7A), but had no signs of diabetes. When the glucose levels were expressed as the area under the curve (AUC), the changes between the two group mice were statistically significant (Figure 7B, p<0.01). Insulin tolerance test (ITT) showed that treatment of GLP-1/*h*IgG2 did not alter the insulin sensitivities (Figure 7C). IPGTT showed that GLP-1/*h*IgG2-treated MDSD mice had improved glucose tolerance (Figure 7D).

## Discussion

GLP-1 has important functions on regulation of glucose homeostasis and thus has been proposed for the treatment of diabetes. Despite its attractive anti-diabetic actions, the therapeutic potential of using native GLP-1 is limited by its short lifetime (<2 min), mainly due to rapid enzymatic inactivation by DPP-IV [10]; [16] and renal clearance [17]. These limitations have continued to fuel attempts to develop more potent long-acting GLP-1 analogs. We report here that, a GLP-1 fusion protein, GLP-1/*h*IgG2, consisting of GLP-1 fused with human IgG Fc, retaines native GLP-1 properties and demonstrates long-acting characteristics. This recombinant GLP-1 fusion protein has anti-diabetic and other beneficial features of GLP-1.

The IgG fusion molecules potentially are large molecular weight homodimers and are not expected to be rapidly cleared by the kidneys, and thus have a substantially longer half-life and better metabolic profiles [18]; [19]. Fc fusion based drugs provide a numbers of advantages and have become credible alternatives to monoclonal antibodies as therapeutics [20]; [21]. GLP-1/*h*IgG2 fusion molecules are produced as homodimers, comprising of two IgG CH2/CH3 chains fused to a pair of GLP-1 molecules with molecular mass of 70 kilo Daltons (Figure 1D) and had a substantially longer half-life. Our pharmacokinetic data showed that that the fusion proteins are detectable a week after a single dose injection in mice. IPGTT showed that the glucoregulatory effects of GLP-1/*h*IgG2 were maintained 8 days after a single dose-injection, suggesting that the detected fusion protein were still biologically active. Although the fusion protein contains a native GLP-1, the GLP-1 in the fusion design is expected to have reduced susceptibility to degradation since such degrading enzymes have a preference for smaller peptides [22]. Indeed, our in vitro stability assay results supported this notion, which is also supported by our previous evidence that *in vivo* expression of GLP-1/*m*IgG-Fc or exendin-4/*m*IgG-Fc fusion proteins achieved equivalent efficacy in mice [12].

Ligand-receptor binding parameters suggest that GLP-1/hIgG2 has high binding affinity to the GLP-1R in INS-1 cells which is comparable to those of exending-4 (Figure 2) as well as those of native GLP-1 [23]. The retained high binding affinity is also suggestive of the fact that the fusion process most likely did not alter the proximal conformation of native GLP-1. It is likely that GLP-1/*h*IgG2 that contains the genetically engineered linker, equivalent to the natural hinge region of human IgG2, may provide flexibility and sufficient spatial structure for appropriate binding of GLP-1 to its receptor [14]. It is of note that the design of the linker sequence of the genetic fusion is considered to be critical for maintenance of peptide activity [21]. A recent study by Picha KM [24] et al. reported that CNTO736, a GLP-1 peptide analog, genetically fused to a Fc portion of IgG has an optimized linker sequence which provided higher activity when compared to other reported fusions of GLP-1 to an IgG1 Fc or albumin [24]; [25]. Our results indicate that, a hinge region of human IgG2 that functions as a linker, provide an optimized binding of fused GLP-1 molecule to its receptor. Furthermore, the dimeric GLP-1, conjugated with an Fc fragment, is expected to increase the ligand avidity since homodimeric GLP-1 can potentially recruit additional GLP-1Rs and amplify intracellular signaling via preformed GPCR dimers/oligomers [26]. The ability of the fusion protein to stimulate insulin secretion in INS-1 cells in a glucose-dependent manner further suggests that the GLP-1 fusion protein retains the biological activities of the native GLP-1.

Using human IgG2 in the fusion protein appears to be advantageous over the use of other subclasses of IgG. Of all human IgG isotypes, IgG2 has the lowest affinity for FcγRI [27]; [28]. FcγRI is a high affinity Fc receptor that binds IgG1, IgG3, or IgG4 in monomeric form, and can induce antibody dependent cellular toxicity (ADCC) [29]; [30]. IgG2-coated red blood cells (RBCs) did not activate phagocytes and were not lysed by these cells, unlike RBCs coated with other IgG isotypes [29]. In contrast to FcγRI, other activating Fc receptors are of low affinity and only bind multimeric IgG as found in immune complexes and, in any case, IgG2 also has low affinity for these receptors [30]. Thus, native IgG2 or GLP-1/*h*IgG2 constructs are not likely to bind to activating Fc receptors in vivo. Moreover, IgG2-Fc binds to the inhibitory Fc-γRIIB receptor on some immune cells [30], which further reduces the probability of Fc induced immunity. The use of *h*IgG2Fc would be a better control, but our previous work showed that Fc fragments have no effect on glucose homeostasis [12], [13]. The tissue distribution of *h*IgG2Fc might be different since it will not bind to GLP-1 receptor positive cells. The only receptors hIgG2Fc is likely to bind are the Fc receptors of immune cells. The relatively low levels of *h*IgG2Fc in control mice would have to compete with mouse IgG, which is present in large amounts in the serum (approximately 10 mg/ml), so that the effect would be minimal or nil. Therefore, we believe that the use of vehicle is an appropriate control in our experiments.

The delivery of protein drugs has often led to the rise in production of neutralizing antibodies which may diminish or abolish the activity of a peptide hormone in the recipient. Neutralizing antibodies are generated mostly when the injected protein is foreign object containing antigenic determinants or when the protein is co-administered with a vehicle or by a route that promotes immunity [31]; [32]. This is initiated when B-lymphocytes bind to the hormone through the B-cell antigen receptor. However, B-cell stimulation can be prevented by co-ligating inhibitory FcγRIIB receptors [33]; [34]. We postulated that B-cell reactivity to GLP-1 will be prevented or diminished when this peptide is fused to an Fc segment, through the co-engagement of the FcγRIIB [13]; [30]. This is supported by our recent observations in mice, where we found that exendin-4 neutralizing antibodies were detected in mice exposed to Ex-4 but not to Ex4-IgG-Fc (Liang YM et al unpublished data), consistent with the tolerance effect of IgG carrier proteins [35]; [36].

Binding of GLP-1 activates the adenylyl cyclase pathway, which ultimately results in a increase of glucose-induced insulin secretion [23]; [37]. Our previous data [13] and others [24] indicated GLP-1-Fc fusions have operated this pathway to exert GLP-1 action in insulin-secreting beta-cells. In present study, we found that GLP-1/*h*IgG2 was rapidly and extensively internalized after binding to GLP-1R in INS-1 cells, representing the characteristics of native GLP-1 upon binding to its counterpart [38]. In addition, the internalization of GLP-1/*h*IgG2 in INS-1 cells was found to be dynamin-dependent activity, since the endocytosis of the GLP-1/*h*IgG2-GLP-1R complexes was significantly blocked in the beta-cells expressing dominant negative dynamin. These results further suggest that, like native GLP-1 [15], GLP-1/hIgG2 initiated GLP-1R trafficking is mediated by a mechanism involving dynamin-caveolin-1 activities in INS- cells[15].

There is potential concern that with long-lived GLP-1R agonists continual exposure of the peptide may result in receptor tachyphylaxis. However, GLP-1-Fc fusion protein did not appear to cause this, at least in mice [24]. Previous *in vivo* studies in rats also showed that a 48-h infusion of GLP-1 resulted in increased insulin secretion and beta-cell proliferation, with no evidence of loss of activity [39]. In addition, mice with transgenic exendin-4 expression displayed significantly increased insulin levels after oral glucose administration [40], suggesting that incretin responses were not suppressed by the continuous presence of exendin-4. Furthermore, clinical studies have demonstrated that while continuous infusion of native GLP-1 in type 2 diabetic patients reduced blood glucose uniformly after 1 or 6 weeks of treatment [41], a comparison of 16-h and 24-h continuous infusion showed that a better glycemic control could obtained with sustained 24-h treatment [42]. Finally, IPGTT results showed that 8 days after a single dose-injection of GLP-1/*h*IgG2 displayed comparable GLP-1 glucoregulatory effects as seen in an acute IPGTT study in CD1 mice. Taken together, these findings highlight the fact that prolonged stimulation of the GLP-1R induces appropriate biological responses. It is possible that the internalization machinery of GLP-1R in the beta-cells may provide a continuous presence of accessible GLP-1R under *in vivo* conditions. Internalization of receptor-ligand complexes is an essential feature of the function of GPCRs and is considered to be required for the dissociation of the ligand from its receptor and for re-sensitization of the receptor [43]; [44]. This process is possibly executed by dephosphorylation of the receptor by phosphatases encountered in the transit through the endosomal compartment [45]; [46].

In summary, we have developed a platform for genetic engineering GLP-1 mimetics, in particular, GLP-1 fused with IgG-Fc segment to achieve long-acting functionality and high efficacy. Various GLP-1 chimera through fusion with either mouse IgG-Fc or human IgG-Fc constructs are designed in order to provide a means for pre-clinical and clinical research. The data presented suggest that the GLP-1 mimetics, exemplified by GLP-1/*h*IgG2, in which native GLP-1 fused with human IgG2-Fc has improved pharmacokinetic and pharmacodynamic profile. It retains natural GLP-1 binding properties and upon binding, it initiates GLP-1-GLP1R complex membrane trafficking to exert GLP-1 actions, including stimulation of insulin secretion from the beta-cells, and bring into play the glucoregulatory and anti-diabetic effects *in vivo*. Our data suggest that GLP-1/*h*IgG2 may find application as a long-lasting GLP-1 analogue.
